# Supplementary material for: Dietary Whey Protein Supplementation Increases Immunoglobulin G Production by Affecting Helper T Cell Populations after Antigen Exposure
Source: Foods. 2021 Jan 19;10(1):194. doi: 10.3390/foods10010194 (PMC7835905; doi:10.3390/foods10010194)
Supplement: Supplementary file 1 [file foods-10-00194-s001.zip › foods-1028898-supplementary.pptx]

## Slide 1
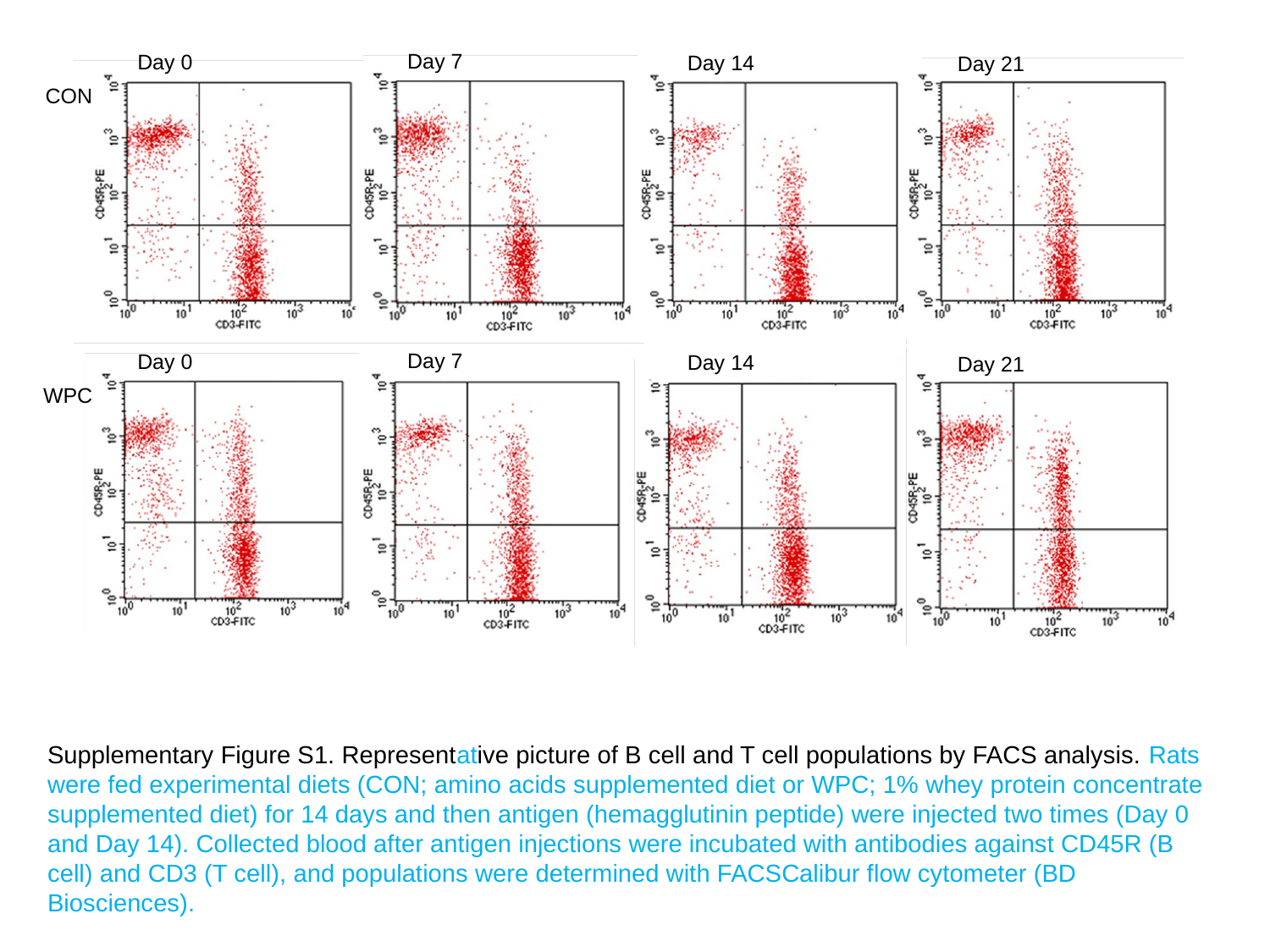

Day 7
Day 0
Day 14
Day 21
CON
Day 7
Day 0
Day 14
Day 21
WPC
Supplementary Figure S1. Representative picture of B cell and T cell populations by FACS analysis. Rats were fed experimental diets (CON; amino acids supplemented diet or WPC; 1% whey protein concentrate supplemented diet) for 14 days and then antigen (hemagglutinin peptide) were injected two times (Day 0 and Day 14). Collected blood after antigen injections were incubated with antibodies against CD45R (B cell) and CD3 (T cell), and populations were determined with FACSCalibur flow cytometer (BD Biosciences).

## Slide 2
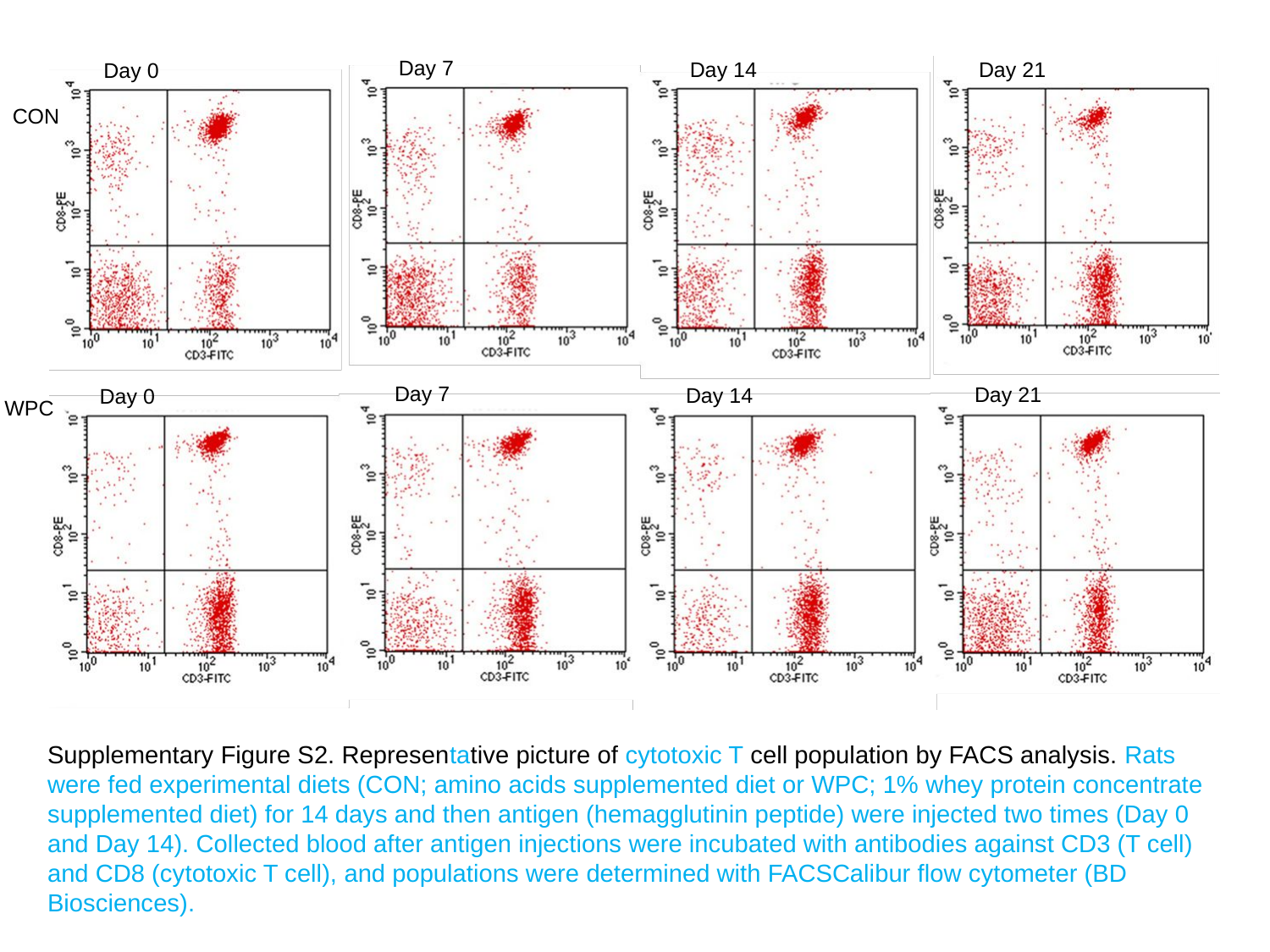

Day 7
Day 21
Day 14
Day 0
CON
Day 7
Day 21
Day 14
Day 0
WPC
Supplementary Figure S2. Representative picture of cytotoxic T cell population by FACS analysis. Rats were fed experimental diets (CON; amino acids supplemented diet or WPC; 1% whey protein concentrate supplemented diet) for 14 days and then antigen (hemagglutinin peptide) were injected two times (Day 0 and Day 14). Collected blood after antigen injections were incubated with antibodies against CD3 (T cell) and CD8 (cytotoxic T cell), and populations were determined with FACSCalibur flow cytometer (BD Biosciences).

## Slide 3
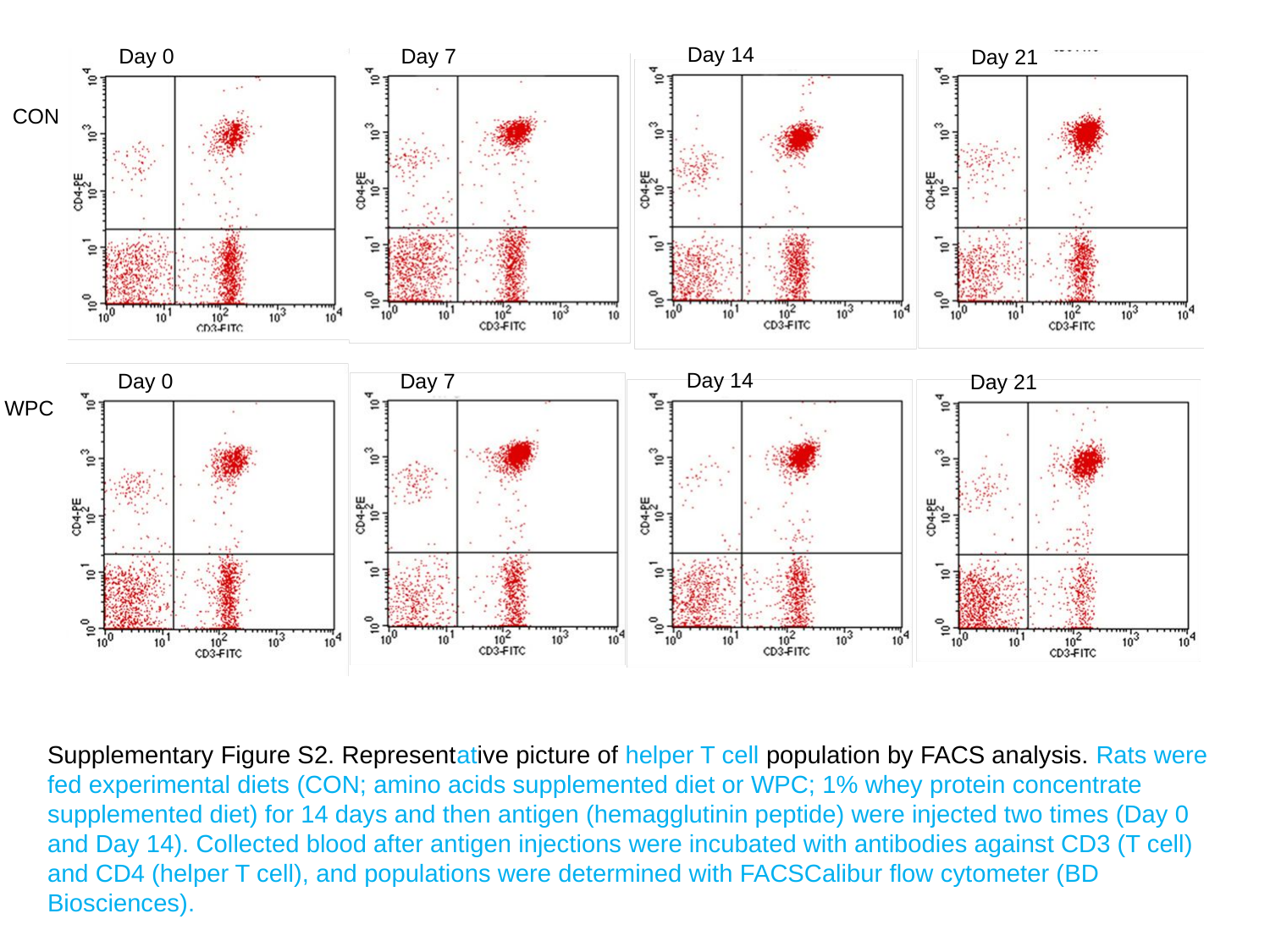

Day 14
Day 7
Day 0
Day 21
CON
Day 14
Day 7
Day 0
Day 21
WPC
Supplementary Figure S2. Representative picture of helper T cell population by FACS analysis. Rats were fed experimental diets (CON; amino acids supplemented diet or WPC; 1% whey protein concentrate supplemented diet) for 14 days and then antigen (hemagglutinin peptide) were injected two times (Day 0 and Day 14). Collected blood after antigen injections were incubated with antibodies against CD3 (T cell) and CD4 (helper T cell), and populations were determined with FACSCalibur flow cytometer (BD Biosciences).
